# Metastatic colorectal carcinoma initially diagnosed by bone marrow biopsy: a case report and literature review

**DOI:** 10.1186/s43046-020-00040-6

**Published:** 2020-07-17

**Authors:** Reham Alghandour, Gehad A. Saleh, Farida Ahmed Shokeir, Mohammad Zuhdy

**Affiliations:** 1 Medical Oncology Department, Oncology Center, Mansoura University, Mansoura, Egypt; 2 Diagnostic Radiology Department, Mansoura University Hospitals, Mansoura, Egypt; 3 Pathology Department, Faculty of Medicine, Mansoura University, Mansoura, Egypt; 4 Surgical Oncology Unit, Oncology Center, Mansoura University, Mansoura, 35516 Egypt

**Keywords:** Colorectal carcinoma, Bone marrow metastasis, Case report, Metastasis

## Abstract

**Background:**

Colorectal carcinoma still represents a global health burden despite the advances in its management. The most common sites of distant metastasis from colorectal carcinoma are hepatic and pulmonary metastases while metastases are rarely reported to affect the bone marrow.

**Case presentation:**

We report a 33-year-old female patient who presented with fever of unknown origin, bone aches limited to the lower back and pelvis, and pancytopenia. She was diagnosed by a bone marrow biopsy as a case of metastatic rectosigmoid carcinoma. Serum tumor markers were within normal ranges; CT, MRI, and colonoscopy confirmed the presence of malignant rectosigmoid mass with bone and ovarian metastases.

**Conclusion:**

Though being rare, bone marrow metastasis should be suspected in colorectal carcinoma cases with abnormalities in peripheral blood count.

## Background

Worldwide, colorectal cancer is ranked third after lung and breast carcinomas among the most commonly diagnosed cancers and the second cause of mortality [1]. Notably, it is ranked seventh among Egyptian males or females [2]. At the time of diagnosis, approximately 20% of colorectal cancer patients present with distant metastasis [3]. Distant metastasis from colorectal carcinoma is most commonly reported in the liver (up to 70%) followed by the lungs (up to 30%) [4], while it was rarely reported to metastasize to the bone marrow [5]. In this report, we present a case of colorectal carcinoma that was initially diagnosed by a bone marrow biopsy.

## Case presentation

**Fig. 1:**
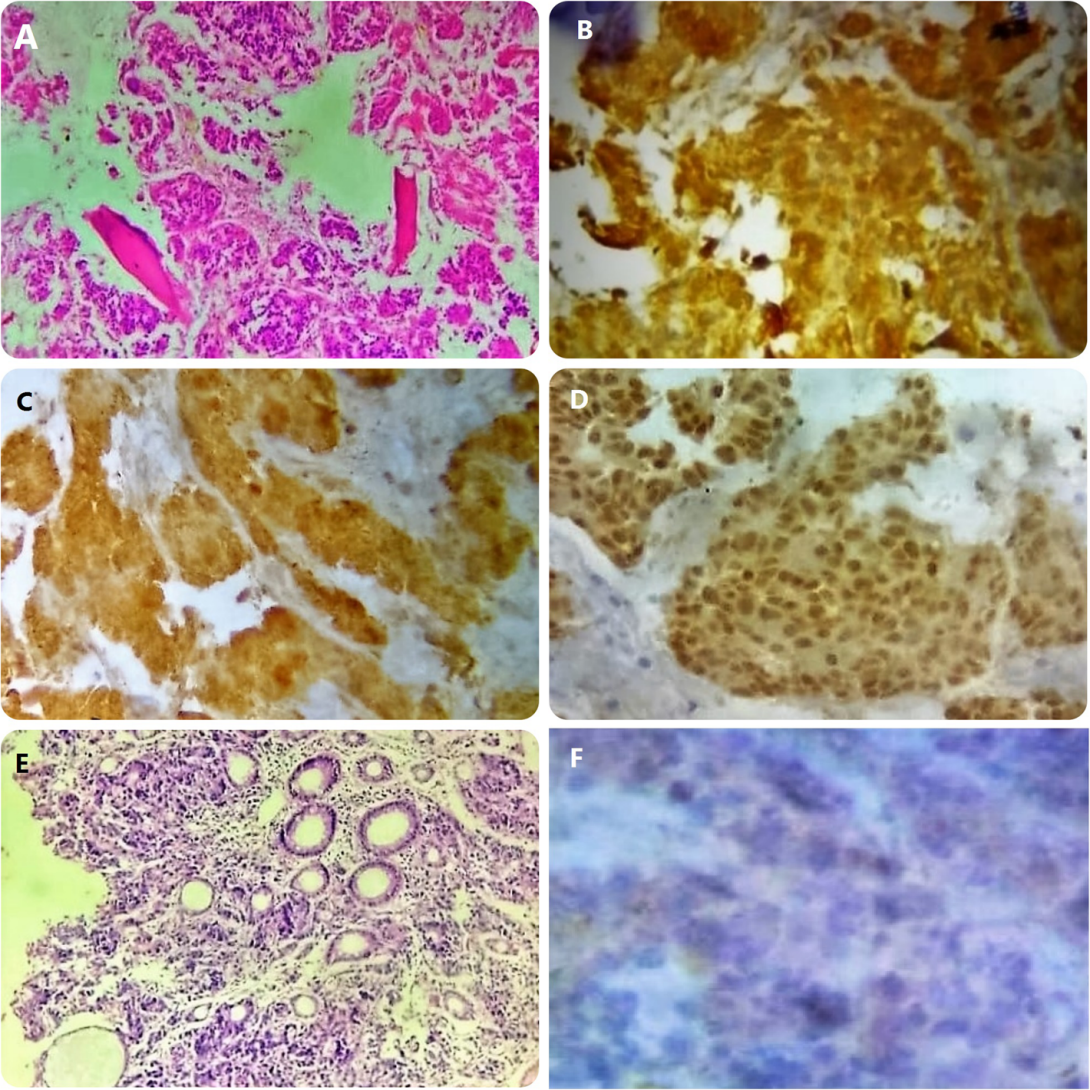
**a**: H&E; BM trabeculae with intervening nests of malignant epithelial cells admixed with foci of necrosis (× 100). **b** Positive cytoplasmic staining of neoplastic cells for CK (× 400). **c** Positive cytoplasmic staining of neoplastic cells for CK20 (× 400). **d** Positive nuclear staining of neoplastic cells for CDX-2 (× 400). **e** Colonoscopic biopsy showing the same malignant proliferation demonstrated in BM, with acinar and sheeting patterns separated by desmoplasia (× 100). **f** Negative staining of neoplastic cells for WT-1 (× 400)

**Fig. 2:**
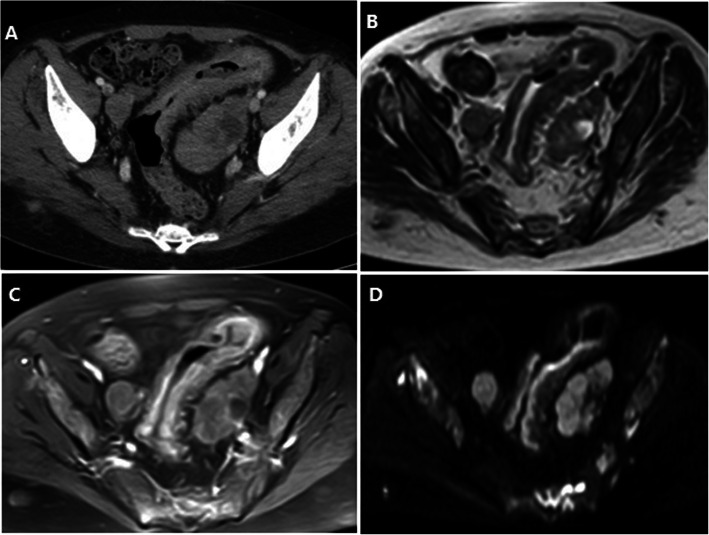
**a** Axial post-contrast CT pelvis, **b** axial T2-weighted image, and **c** post-contrast T1-weighted image revealed diffuse asymmetrical circumferential thickening of the rectosigmoid with stranding of related fat planes and bilateral predominantly solid complex heterogeneously enhanced ovarian masses, larger on the left side (Krunkenberg tumor). **d** Axial diffusion-weighted image (DWI) revealed multiple diffusion restricted infiltrative bony deposits at both the iliac bones and sacrum without cortical destruction (missed on CT scan) with better delineation of the diffusion-restricted malignant rectosigmoid infiltration and bilateral Krukenberg tumors

A 33-year-old female patient was referred to our center at Mansoura Fever Hospital with a history of pyrexia of unknown origin of 3 weeks duration and complete blood count showing pancytopenia. White blood cell count was 3.2 k/μl, red cell count was 2.87 M/μl, hemoglobin level 7.8 g/dl, and platelet count was 25,000/μl. The mean corpuscular volume (MCV), mean corpuscular hemoglobin (MCH), and mean corpuscular hemoglobin concentration (MCHC) were within the normal range denoting normocytic normochromic anemia. Erythrocyte sedimentation rate (ESR) was 70 and 110 at the first and second hours, respectively. Serum ferritin was markedly elevated 4000 ng/ml (normal range 13–400 ng/ml), and serum lactate dehydrogenase was reported to be high 4878 U/L (normal range 100–190 U/L). Antinuclear antibodies (ANA), anti-ds-DNA, and direct and indirect Coombs tests were all negative. Hepatitis B and C and HIV viral markers were all negative. Widal agglutination, *Brucella* microagglutination, and *Helicobacter* antigen in stools tests were performed, and their results were negative. Plain chest radiography and abdominal sonography were unremarkable. After being admitted, a detailed history was retrieved from the patient where she reported bone aches limited to the lower back and the pelvis, vomiting, and diarrhea. Examination revealed no organomegaly and no lymphadenopathy. Urine, sputum, blood cultures, bone marrow aspirate, and biopsy were requested on the second day of her admission, and the aspirate revealed hypocellular bone marrow infiltrated by non-hematopoietic cells. That is why the computerized tomographic scan of the chest, abdomen, and pelvis and serum tumor markers were ordered. Serum carcinoembryonic antigen (CEA) was significantly elevated 17 ng/ml, while other markers were within the normal range. The report of examination of pathology slides from the bone marrow was reported 10 days later and revealed adequate bone marrow spaces showing infiltration by malignant tumoral proliferation arranged mainly into sheets and nests separated by desmoplasia. These were lined by malignant epithelial cells that were large, pleomorphic with high N/C ratio, moderate atypia, and foci of necrosis. Immunohistochemical stains (IHC) were performed including pan-cytokeratin (CK), CK7, CK20, Wilms’ tumor-1 (WT-1), and Caudal Type Homeobox 2 (CDX2) for the possibility of primary ovarian versus colonic origin. Neoplastic cells were diffusely positive for pan CK, CK20, and CDX2 supporting gastrointestinal origin (Fig. 1a–d). CT scan revealed rectosigmoid mural thickening with a left ovarian complex lesion; magnetic resonance imaging (MRI) was recommended for better characterization. MRI revealed a malignant rectosigmoid infiltrative lesion with bilateral ovarian masses mostly Krukenberg tumor as well as infiltrative bony deposits at both the iliac bones and the sacrum. No other metastases were detected by the radiological workup (Fig. 2). Colonoscopy was performed a week after the admission and revealed a typically malignant rectosigmoid stenosing growth at 12 cm from the anal verge that was biopsied. The result of the histopathological examination of the biopsy was revealed after 10 days and confirmed the presence of a malignant tumoral proliferation that matched the same morphology presented initially in BM (Fig. 1e, f). The patient was admitted to the isolation ward, and broad-spectrum antibiotics were initiated until the results of the culture and sensitivity tests were revealed. Unfortunately, she succumbed due to sepsis 1 week after her diagnosis.

## Discussion

Hereby, we report a female patient who presented with bone marrow metastasis from rectosigmoid carcinoma and was diagnosed with pancytopenia.

Globally, colorectal cancer still represents a major health burden despite the recent advances in its management. This is mainly attributed to its pattern of spread and metastasis [6]. The most commonly reported solid malignancies that could metastasize to the bone marrow are breast or gastric or prostatic carcinomas [7], whereas colorectal carcinoma was rarely reported in the literature to be complicated by bone marrow metastasis [8]. To the best of our knowledge, twenty-one cases of bone marrow metastasis were previously reported in the literature. Table 1 summarizes their characteristics, management, and follow-up data.

**Table 1:** Summary of the demographics and clinical data of previously reported cases of bone marrow metastasis from colorectal cancer

Article	Age/sex	Presentation pattern	Peripheral blood count	Primary tumor site	Other metastases	Treatment	Survival
Yoshioka et al. [9]	62/M	Primary	DIC	Rectum (diagnosed by autopsy)	N/A	N/A	Died after 13 days
Sema et al. [10]	61/M	N/A	DIC	Sigmoid	No	Supportive	Died after 2 weeks
Lee et al. [11]	67/M	Primary	Anemia/thrombocytopenia	Hepatic flexure	Bone	FOLFOX	Alive 18 weeks after diagnosis
Huang et al. [12]	79/M	Primary	DIC	Rectum	N/A	5FU + leucovorin	Died after 83 days
Pleyer et al. [13]	48/M	Primary	Thrombocytopenia	N/A	Peritoneal/pleural/pericardial effusion/mediastinal lymphadenopathy	FOLFOX + bevacizumab (surgery for the primary)	Died after 5 cycles of chemotherapy
Misawa et al. [14]	51/M	Primary	DIC	Ascending colon	Bone	No	Died after 25 days
Wang et al. [15]	37/M	Primary	Anemia/thrombocytopenia	Sigmoid	Bone	FOLFOX + cetuximab	Died after 3 months
Isozaki et al. [16]	45/M	Primary	DIC	Descending colon	Lymph nodes	mFOLFOX	N/A
Song and Dwyre [17]	70/M	Primary	Anemia	Rectum	Bone	N/A	N/A
Orgel et al. [18]	65/F	Primary	MAHA	Sigmoid	Hepatic	FOLFOX + cetuximab	Died after 7 months
Naito et al. [19]	61/M	Primary	DIC	Transverse colon	Bone/lymphadenopathy	XELOX + bevacizumab	Resolution after 4 cycles of chemotherapy + resection was considered
Nakashima et al. [20]	65/M	Primary	DIC	Rectum	Bone	mFOLFOX + bevacizumab + surgery	Died 128 POD
Shah et al. [8]	58/M	Primary	DIC	Cecum	Hepatic/mediastinal lymphadenopathy	mFOLFOX + FOLFIRI + bevacizumab	Died after 6 months
Van Banderin et al. [21]	65/F	Primary	DIC	Sigmoid	Bone	XELOX	Died after 8 months
Lim et al. [5]	74/F	Recurrent	Anemia/thrombocytopenia	History of right hemicolectomy	No	No	10 days after diagnosis
Assi et al. [6]							
1st case	75/M	Recurrent	Anemia/leucopenia	Rectum	No	FOLFOX + bevacizumab	Alive after 6 months
2nd case	56/M	Primary	Anemia/thrombocytopenia	Rectosigmoid	Bone	FOLFOX	Died after 6 months
3rd case	55/M	Primary	Anemia/thrombocytopenia	Ascending colon	Bone	FOLFOX	Died after 4 months
Hanamura et al. [22]	60/M	Primary	DIC	Sigmoid	Bone	mFOLFOX + CapeOx + irinotecan + pantumumab	Died after 10 months
Takeyama et al. [23]	65/M	Recurrent	Leucopenia/thrombocytopenia	Hx of rectal resection	Bone/lung	mFOLFOX	Died 263 days from meningeal metastasis
Zeeneldin et al. [24]	42/M	Primary	Anemia/thrombocytopenia	Rectum	Bone/lung/lymphadenopathy	XELOX	Died after 6 months

*DIC* disseminated intravascular coagulopathy, *MAHA* microangiopathic hemolytic anemia, *N/A* not available, *POD* postoperative day

In an autopsy study, Weiss et al. reported that 24% of colorectal cancer patients had isolated bone marrow metastasis, while patients who suffered from either metastasis in the bone marrow and liver or bone marrow, liver, and lung to be 16% and 34%, respectively [25]. In the current report, the patient suffered from ovarian metastasis in addition to the bone marrow metastasis.

In the present case, bone marrow metastasis was the first presentation of rectosigmoid cancer. Two hypotheses were postulated to explain the rarity of this presentation. Firstly, bone marrow metastasis is never the only apparent site of distant metastasis of solid malignancies. Secondly, the clinical significance of bone marrow studies is minimal except if abnormalities in peripheral blood count existed [6]. Several factors could explain the tendency of solid tumors to metastasize to the bone marrow. They included the abundant vascularity, slow blood flow, and the interactions between the bone marrow stroma and tumor cells that lead to the release of growth factors. In our case, the metastases were encountered in the sacrum and iliac bones that were previously linked in the literature to the paravertebral venous plexus of Baston due to its valveless communications [26].

Previous studies reported an 18% incidence of pancytopenia in patients with bone marrow metastasis, while the incidence of bicytopenia, anemia, neutropenia, or thrombocytopenia was found to be 32%, 68%, 23%, and 58%, respectively [27, 28]. Other cases presented with disseminated intravascular coagulopathy (DIC), microangiopathic hemolytic anemia (MAHA), or thrombocytopenic purpura [8].

Cytopenias encountered as a consequence of bone marrow metastasis could increase the risk of bleeding and infection and importantly delay the administration of chemotherapy and targeted therapy or even prevent their delivery. Patients who suffer from bone marrow metastasis experience poor survival ranging from 5 to 7 months. Survival is mainly affected by some factors including the presence of other metastasis, platelet count, and the patient’s performance status [27]. Unfortunately, our case died 1 week after diagnosis due to overwhelming sepsis.

Viehl et al. in their prospective trial found that 38% of stage I–III colon cancer patients could have bone marrow micrometastases (BMM). They concluded that BMM are independent prognostic factors for both disease-free survival (DFS) and overall survival (OS); however, the clinical significance of BMM is still debatable [29].

## Conclusion

Though being rare, bone marrow metastasis should be suspected in cases who presented with abnormalities in peripheral blood count. Once a diagnosis is reached, rapid and appropriate treatment should be initiated to defeat the inevitable deterioration of the disease.

## Data Availability

All data generated or analyzed during this study are included in this published article.

## Abbreviations

ANA: Antinuclear antibodies
BM: Bone marrow
BMM: Bone marrow micrometastasis
CDX2: Caudal type homeobox 2
CEA: Carcinoembryonic antigen
CK7, CK20: Cytokeratin 7,20
CT: Computed tomography
DFS: Disease-free survival
DIC: Disseminated intravascular coagulopathy
ESR: Erythrocyte sedimentation rate
IHC: Immunohistochemical stains
LDH: Lactate dehydrogenase
MAHA: Microangiopathic hemolytic anemia
MCH: Mean corpuscular hemoglobin
MCHC: Mean corpuscular hemoglobin concentration
MCV: Mean corpuscular volume
MRI: Magnetic resonance imaging
OS: Overall survival
WT-1: Wilms’ tumor-1
